# Exploring the Role of the Food Environment on Food Shopping Patterns in Philadelphia, PA, USA: A Semiquantitative Comparison of Two Matched Neighborhood Groups

**DOI:** 10.3390/ijerph10010295

**Published:** 2013-01-14

**Authors:** Jana A. Hirsch, Amy Hillier

**Affiliations:** 1 Center for Social Epidemiology and Population Health, University of Michigan School of Public Health, 2675 SPH I, 1415 Washington Heights, Ann Arbor, MI 48109, USA; 2 University of Pennsylvania School of Design, 210 South 34th Street, Philadelphia, PA 19104, USA; E-Mail: ahillier@design.upenn.edu

**Keywords:** food environment, supermarket, shopping behavior, methodology, Geographic Information Systems (GIS)

## Abstract

Increasing research has focused on the built food environment and nutrition-related outcomes, yet what constitutes a food environment and how this environment influences individual behavior still remain unclear. This study assesses whether travel mode and distance to food shopping venues differ among individuals in varying food environments and whether individual- and household-level factors are associated with food shopping patterns. Fifty neighbors who share a traditionally defined food environment (25 in an unfavorable environment and 25 in a favorable environment) were surveyed using a mix of close- and open-ended survey questions. Food shopping patterns were mapped using Geographic Information Systems (GIS). Stores visited were beyond the 0.5-mile (805 meters) radius traditionally used to represent the extent of an individual’s food environment in an urban area. We found no significant difference in shopping frequency or motivating factor behind store choice between the groups. No differences existed between the two groups for big food shopping trips. For small trips, individuals in the favorable food environment traveled shorter distances and were more likely to walk than drive. Socioeconomic status, including car ownership, education, and income influenced distance traveled. These findings highlight the complexities involved in the study and measurement of food environments.

## 1. Introduction

Over the past decade there has been a surge in research investigating the effect of neighborhood food environment and nutrition-related health outcomes [1,2,3]. There is significant evidence to suggest that supermarket accessibility and density are associated with weight status and health outcomes [4,5,6,7,8,9,10], although one recent longitudinal study did not find these associations [11]. A smaller amount of literature has focused on within-store food availability, including price and quality [12]. While the field has advanced significantly towards determining the effect of the built food environment, several methodological and conceptual problems continue to limit advancements in understanding this complex problem.

Three recent reviews found that the spatial approaches used to measure the environment have been limited to densities or “buffers” around a residence (*i.e.*, 0.5 mile or 805 meters radius), distance to a closest outlet, or administrative boundaries (such as census tracts or block groups) or “buffer” [2,13,14]. This “zone-based aggregate spatial framework” may set arbitrary boundaries in the built food environment and implies that individuals recognize these boundaries and conduct their daily lives restricted to them [2,15,16,17].

In order to better capture the environment in which individuals live, work, shop, and play, “activity spaces” have been suggested as an alternative to these zone-based or residentially focused measures [5,17,18,19]. Despite advances in technology that would allow for more complex modeling of geographic access, limited research utilizes these tools [20,21,22,23,24]. To date, three studies [18,25,26] have used global positioning systems (GPS) or travel diaries, Geographic Information Systems (GIS) and the concept of activity space to investigate the role of the built food environment on health behavior and health outcomes. Their findings demonstrated that traditional food environment measures may be poor proxies for the actual environmental exposure of individuals as activity spaces were larger than the traditional neighborhoods and only weakly associated with the traditionally defined environmental features of residential neighborhoods [18]. Significantly more research needs to be done that takes advantage of the existing technology. However, the cost of executing these methods, in combination with the heavy burden on participants, restricts the feasibility of their widespread use.

Yet even the use of GPS technology and activity spaces cannot account for, or incorporate, the role of individual choice. Current methodologies overlook consumer travel patterns and individual selection of shopping destinations [19,21,27,28]. By doing so, research has been unable to elucidate the mechanism by which the environment affects behavior and thus health outcomes [29]. Qualitative research that captures patterns of shopping habits is necessary to not only understand the contexts in which individuals interact with their environment but also to tease apart the complex path between the physical food access and health outcomes.

This article combines GIS, descriptive statistics, regression models, and qualitative analysis to analyze survey data on food shopping patterns and the perception of residents’ food environments. We aim to answer two research questions: (1) Is there a difference in travel mode and distance to food shopping venues among individuals in high and low quality food environments? and (2) What are some of the individual and household-level factors associated with food shopping patterns? Ultimately, this article aims to explore some of the individual complexities necessary to conceptualize and measure the food environment. Finally, we hope to raise questions about the current methodological reliance on zone-based aggregate spatial framework and the eclipse of personal choice when discussing the food environment.

## 2. Experimental Section

### 2.1. Block Selection

Two matching street face blocks in Philadelphia were chosen to represent distinct food environments based on proximity to major food sources. Using ArcView GIS 9.3 software (ESRI Redlands, CA, USA), block groups from the 2000 U.S. Census were classified as being in either a favorable or unfavorable food environment, based on whether they were within or not within 0.5 miles (approximately 805 meters) of a chain supermarket as identified by a retail database from TradeDimension. Block groups were excluded if they were not predominantly residential (defined as more than 2% commercial) and if residents were not mainly English-speaking (non-Hispanic), due to the survey language. One block group from each food environment classification was non-randomly selected based on racial and income characteristics. Due to the disproportionate allocation of chain supermarkets by neighborhood sociodemographic characteristics, and the varying ethnic composition of different racial groups across the city, the block groups chosen were predominantly white, middle- to upper-income areas with equal population and household densities. While much of the emphasis in food environment research is on low-income, minority communities, the use of white, middle- to upper-income block groups allowed for a better match of neighborhood characteristics, and reduces the potential for confounding by ethnic groups who may seek out specialty or ethnic food stores. Finally, within each block group, two street face blocks with similar types and amount of housing were identified by a site visit.

One street face block is located in West Philadelphia and is 0.4 miles (644 meters) from a full-service discount supermarket, 0.5 miles (805 meters) from a large chain supermarket, 0.2 miles (322 meters) from a weekly farmers’ market, 0.3 miles (483 meters) from a locally-sourced specialty grocer, 0.4 miles (644 meters) from an international grocer, and 0.6 miles (966 meters) from a food coop (many major food sources nearby). The housing stock is primary single-family row houses with a few apartments. For this paper we will refer to this block as the “favorable food environment”. The other street face block, in the Fairmount section of Philadelphia, is 0.1 miles (161 meters) from a chain convenience store that sells gas. The nearest supermarket is 1.0 miles (1,609 meters) away (no major food sources nearby). There is a seasonal farmers’ market located 0.7 miles (1,126 meters) away that was not open during the course of this study. Housing in this street segment is also a mix of row houses and apartment buildings. For this paper we will refer to this block as the “unfavorable food environment”. While these block groups were matched on racial composition, income, population, number of houses, housing type, family size, and vacancy, the West Philadelphia block group consisted of a younger population (greater percentage of the population in 2000 Census between 18–21 and 22–29) and a higher percentages of households being renter rather than owner occupied. These differences are anticipated given the close proximity of the West Philadelphia block to the University of Pennsylvania campus.

### 2.2. Survey Methodology

Researchers went door-to-door visiting every house on the two blocks up to four times between 14 February 2010 and 14 March 2010. Researchers asked to speak to the primary food shopper of the household who was 18 years of age or older or the secondary food shopper if the primary food shopper was not available. Researchers administered a 58-item survey about food shopping, perception of the food environment, and factors affecting store choice.

Participant sociodemographic characteristics were assessed using questions derived from the Census on number of people in the household, owner- or renter-occupied unit, race, Hispanic ethnicity, employment status, and education. Additional questions were added on the number of cars owned (one, more than one, we only use car share, we don’t drive), length of time at the current address (less than a year, between 1 and 2 years, between 2 and 5 years, between 5 and 10 years, more than 10 years), neighborhood safety (very safe, safe, not very safe, not safe at all, varies by time of day), self-rated health, functional limitations (“During the past 4 weeks, were you limited in work or other activities you ordinarily do as a result of your physical health?”), and participation in the Supplemental Nutrition Assistance Program (SNAP).

Since no standard survey tool has been validated for collecting food shopping behavior [30], researchers designed a sequence of questions about both big and small food shopping patterns, including location, frequency, mode of transportation, and justification for store choice. Big food shopping was defined verbally to participants as a trip in which the participant spends $100 or more, or in which they buy enough food to completely fill one grocery cart or more. Small food shopping was defined as a trip in which the participant spends $40 or less, or in which they buy only enough food to fill two or fewer grocery bags. Participants were asked to identify and provide an intersection for the stores in which they do their shopping trips (“For those big/small food shopping trips, how many different stores, stands, or farmers’ markets do you regularly visit? Keep in mind we will be asking for the name and address of these stores”. If more than zero, then asked “For those big/small food shopping trips, what is the name and address of the (first) store you normally shop at?”). Participants could list one store for big food shopping and up to three for small food shopping trips. To assess reasons for choosing specific food stores, participants were given the opportunity to answer an open-ended question (“Why do you go to this specific store?”) and were asked to rank how important a given factor was for their household when choosing a food store (“Please rate (1–5) how important these factors are for your household in choosing a food store with one being not at all important, and five being very important”. Assessed for: distance to store, quality of items, price, store atmosphere, and selection).

Questions regarding perception of the food environment were taken from Freedman and Bell [31] in which participants were asked to rate food store access and quality in their neighborhood according to a five-point Likert scale. Questions included ease of buying fresh fruits and vegetables, stores having almost everything necessary on a weekly basis, sale of healthy foods, preference for shopping at the local store, price of the local stores, or local stores stocking outdated or rotten products. Each answer was then assigned values 1 through 5, with rotten products reverse scored, and the average of the questions was taken to get a mean food environment score for each participant, with higher scores representing the perception of more food access.

Researchers used HP iPAQ 110 PDAs programmed with Pendragon software to record the interview data. Participants were consented verbally to maintain anonymity and residences in which a survey was completed were recorded separately from the surveys to maintain anonymity and avoid duplicate households. This study protocol was approved by the University of Pennsylvania Institutional Review Board.

### 2.3. Analysis of Survey Data

The location of stores where participants reported shopping were geocoded using ArcView GIS 9.3 software and a 2008 street centerline file from the City of Philadelphia. All participants in the same block were assigned the center point of their block as a home address. Euclidean distance between the centroid of the block and the stores where they shop was calculated using PointDistance version 9 script for ArcMap (City of Scottsdale GIS Department, 2009).

Survey results were analyzed using SAS Software^®^, v.9.2 (SAS Institute, Inc., Cary, NC, USA). Descriptive statistics were calculated for demographic, food shopping patterns, and perceived food access for each block. Fisher’s exact test statistics were calculated to examine differences between participants in the two different food environments. Mean distances for big and small food shopping in each block were compared using paired *t*-tests. Distances traveled (in miles) for big and small shopping were analyzed relative to individual-level characteristics using generalized linear models. Covariates included in the model were car status (one, more than one, we only use car share, we don’t drive), education (less than high school, high school or equivalent, some college/vocational training, college graduate, professional/graduate degree), income (less than $20,000, $20,000–$50,000, $50,000–$100,000, More than $100,000), employment (full-time, part-time, retired, student, unemployed not seeking, unemployed seeking), total number of individuals in the household including roommates (continuous), and block (as a proxy for food environment). Responses to open-ended questions on store choice were analyzed using qualitative coding of common themes and keywords by a priori codes, word repetition and grounded codes.

## 3. Results and Discussion

Researchers successfully contacted 77 out of 103 (61 percent) of households. Of those contacted, 25 from each block completed the survey (65 percent) and 17 households (35 percent) refused to participate. While the contact and response rate are low, participant characteristics within these street face blocks were similar to those of the entire block groups in regards to racial composition, average family size, home ownership, and age based on 2000 Census data. The participants on the blocks were comparable to each other in household size, car ownership, home ownership, safety, race, citizenship, education levels, incomes, and self-reported health (see Table 1). Differences between the blocks existed only for length of residence in the neighborhood and employment status. The favorable food environment had 11 participants (44 percent) who had lived in their current home less than one year while the unfavorable food environment block had 13 participants (52 percent) who had lived in their home for more than ten years (*p *= 0.018). Employment status differences were marginally significant (*p *= 0.054) with a larger number of students in the favorable food environment. This explains differences seen in residence time, as 86 percent of students in our sample reported living in their residence less than one year.

**Table 1:** Individual and household characteristics of participants by residential block and food environment (n = 50).

		Unfavorable: no major food stores (½-mile)	Favorable: many major food sources (½-mile)	*p*-value ^a^
**Sample Size (n)**		**25**	**25**	
**Mean household size (SD ^b^) ^c^**		2.92 (1.29)	3.08 (1.41)	*p *= 0.6774
	Mean number adults (SD)	2.16 (0.90)	2.52 (1.36)	*p *= 0.2752
	Mean number of children (SD)	0.76 (1.05)	0.56 (0.71)	*p *= 0.4355
**Car Ownership**				
	We don’t drive	16%	12%	
	We only use car share	0%	8%	
	One	48%	48%	
	More than one	36%	31%	*p *= 0.7237
**Home Ownership**				
	Rental Unit	24%	36%	
	Owner Occupied	76%	64%	*p *= 0.5380
**Length of time in current residence**				
	Less than a year	8%	44%	
	Between 1 and 2 years	0%	0%	
	Between 2 and 5 years	28%	24%	
	Between 5 and 10 years	12%	12%	
	More than 10 years	52%	20%	*p *= 0.0178 *
**How safe feel in neighborhood**				
	Very Safe	12%	20%	
	Safe	56%	64%	
	Varies by time of day	32%	16%	
	Not very safe	0%	0%	
	Not safe at all	0%	0%	*p *= 0.4155
**Race**				
	Black	12%	4%	
	White	68%	84%	
	Asian/South Asian/Pacific Islander	4%	4%	
	AIAN ^d^	0%	0%	
	Black and White	12%	4%	
	White and AIAN	0%	4%	
	Other	4%	0%	*p *= 0.5605
**Hispanic/Latino**		0%	8%	*p *= 0.4898
**US Citizens**		100%	92%	*p *= 0.4898
**Employment**				
	Full-time employment	56%	68%	
	Part-time employment	16%	4%	
	Unemployed, actively seeking	4%	0%	
	Unemployed, not actively seeking	8%	4%	
	Retired	12%	0%	
	Student	4%	24%	*p *= 0.0538
**Education**				
	Less than high school	0%	0%	
	High school or equivalent	8%	0%	
	Some college/associates	20%	16%	
	Bachelors	40%	28%	
	Graduate or professional degree	32%	56%	*p *= 0.2490
**Income**				
	Less than $20,000	16%	16%	
	$20,000–$50,000	28%	20%	
	$50,000–$100,000	24%	16%	
	More than $100,000	32%	48%	*p *= 0.6956
**Self-Reported Health**				
	Poor	0%	0%	
	Fair	8%	0%	
	Good	28%	12%	
	Very Good	28%	48%	
	Excellent	36%	40%	*p *= 0.2139

^a ^Fisher’s exact or *t*-test used to get *p*-value for favorable compared to unfavorable environment. ^b ^SD is Standard Deviation. ^c ^Total number of individuals in the household including roommates. ^d ^AIAN is American Indian or Alaskan Native. * *p *< 0.05.

### 3.1. Food Environment Perceptions

Participants in the two blocks had perceptions of their food environment that matched the GIS-based categorization of unfavorable and favorable food environment and were distinct from those found in the other block. Participants in the unfavorable food environment had a lower mean food environment score of 2.41 (SD 0.74) compared to those in the favorable food environment who had a mean score of 3.52 (SD 0.42) (*p* < 0.0001). Differences existed between the groups for ease of buying fresh fruits and vegetables (*p* < 0.0001), the stores having almost everything necessary on a weekly basis (*p* < 0.0001), and the sale of healthy foods (*p* < 0.0001). No significant differences existed between the groups on preference for shopping at the local store (*p *= 0.2055), price of the local stores (*p *= 0.8075) or the local stores stocking outdated or rotten products (*p *= 0.7018). 

### 3.2. Food Shopping Patterns

There were no significant differences in the frequency of trips made for big or small food shopping across participants from the two blocks (Table 2). However, transportation patterns for big and small food shopping were markedly different. For big food shopping trips, a majority of participants from both blocks (79.1%) drove to the store while for small food shopping trips, walking (41.5%) was more common than driving (32.0%). Transportation mode for big food shopping trips was not significantly different between the two blocks, but for small food shopping trips, the difference was significant (*p *= 0.0004). A majority of participants in the favorable food environment reported walking for their small food trips while a majority of those in the unfavorable food environment reported driving. Although transportation modes varied between the blocks, reasons motivating the choice of transportation type did not. Participants from both blocks reported choosing driving for reasons such as “size of purchase”, “whether I am out doing other errands with the car”, or “transportation type changes based on who is going”.

**Table 2:** Food shopping patterns for big and small food shopping trips by block of residence (n = 50).

		Both street segments combined	Unfavorable: no major food stores (½-mile)	Favorable: many major food sources (½-mile)	*p*-value ^a^
**BIG FOOD SHOPPING TRIPS (n)**		**39**	**21**	**18**	
**Frequency of shopping trips**					
	Never	22.0%	16.0%	28.0%	
	Every month	20.0%	20.0%	20.0%	
	Every two weeks	22.0%	20.0%	24.0%	
	Every week	22.0%	32.0%	12.0%	
	More than once a week	4.0%	0.0%	8.0%	
	Other	10.0%	12.0%	8.0%	*p *= 0.4360
**Distance to store in miles**					
Mean, (SD ^b^) Median		3.02 (3.08) 2.13	2.50 (1.29) 2.40	3.59 (4.25) 1.83	*p *= 0.3110
**Mode of transportation to store**					
	Walk	7.0%	4.2%	10.5%	
	Drive	79.1%	70.8%	89.5%	
	Bicycle	4.7%	8.3%	0.0%	
	Public Transportation	4.7%	8.3%	0.0%	
	Other	4.7%	8.3%	0.0%	*p *= 0.2735
**Why chose mode of transportation**					
	Distance	26.7%	29.7%	21.7%	
	Convenience	35.0%	29.7%	43.4%	
	Cost	8.3%	10.8%	4.3%	
	Weather	8.3%	8.1%	8.6%	
	Children accompanying	15.0%	13.5%	17.4%	
	Other	8.3%	8.1%	8.7%	*p *= 0.8498
**Time of shopping trip**					
	Morning (prior to 12 pm)	20.5%	14.3%	27.8%	
	Early Afternoon (12 pm to 3 pm)	20.5%	23.8%	16.7%	
	Late Afternoon (3 pm to 5 pm)	33.3%	33.3%	33.3%	
	Evening (5 pm to 8 pm)	23.1%	28.6%	16.7%	
	Night (after 8 pm)	2.6%	0.0%	5.6%	*p *= 0.6812
**Pattern of shopping**					
	Completely separate trip	64.1%	71.4%	55.6%	
	On the way to/from work	5.1%	4.8%	5.6%	
	As part of a chain of several errands	25.6%	14.3%	38.9%	
	Other	5.1%	9.5%	0.0%	*p *= 0.1918
**SMALL FOOD SHOPPING TRIPS (n)**		113	52	61	
**Frequency of shopping trips**					
	Never	2.0%	0.0%	4.0%	
	Once a month	6.0%	12.0%	0.0%	
	A few times a month	2.0%	4.0%	0.0%	
	Once a week	32.0%	32.0%	32.0%	
	A few times a week	54.0%	48.0%	60.0%	
	Every day	4.0%	4.0%	4.0%	*p *= 0.4360
**Distance to store in miles**					
	Mean, (SD) Median	1.10 (1.17) 0.93	1.53 (1.34) 1.38	0.73 (0.85) 0.35	*p *= 0.0003 **
**Mode of transportation to store**					
	Walk	41.5%	12.9%	54.6%	
	Drive	32.0%	40.0%	24.7%	
	Bicycle	11.6%	8.6%	14.3%	
	Public Transportation	15.0%	24.3%	6.5%	*p *= 0.0004 **
**Why chose mode of transportation**					
	Distance	28.4%	26.2%	30.1%	
	Convenience	42.8%	40.8%	45.1%	
	Cost	5.2%	2.9%	7.7%	
	Weather	10.3%	12.6%	7.7%	
	Children accompanying	6.2%	7.8%	4.4%	
	Other	7.2%	9.7%	4.4%	*p *= 0.2662

^a^ Fisher’s exact or *t*-test used to get *p*-value for favorable compared to unfavorable environment. ^b ^SD is Standard Deviation. * *p *< 0.05; **p < 0.01.

Reasons for shopping at a particular store fell into seven broad categories for participants from both blocks (see Table 3). Overall, participants in the two blocks cited similar priorities for choosing a store including distance, price, and store atmosphere. Residents in the unfavorable food environment placed greater importance of quality of items (*p *= 0.08) and selection (*p *= 0.08) than residents of the favorable. Geographic location was cited most often as the justification for choosing a specific store. Although most were in relation to residential location, such as “(it is) the only grocery store nearby” and “(it is) right by my house”, participants also discussed proximity to other geographic areas with statements like “(it is) close to my work” or “I go there after getting my kids from their school”. Often respondents discussed convenience, stating “it’s convenient” or “the most convenient for the biggest selection”, although it was unclear whether convenience was derived from distance, time, or another unnamed factor. Cost played an important role, with justifications of store choice by the opportunity to buy “stuff in bulk” or to get items at “great prices”. Selection was incorporated into statements such as “it has everything I want”, “(it) has things I can’t get elsewhere”, and “the selection is unmatched”. A number of participants cited social interaction, social engagement, or social movements as justifications for their choice of stores. Some stated “it is entertaining (to shop here)”, or that “it’s a social place to see my neighbors”. Alternatively, many cited nutritional or political convictions such as the organic or local food movements as driving their store choice. This may reflect the higher socioeconomic status of respondents.

**Table 3:** Qualitative justifications for choosing stores, grouped into seven broad categories.

Category	Description	Examples	Count
Convenience	Specifically referred to convenience	“it’s convenient” “convenient for other purchases” “has convenient parking” “the most convenient for the biggest selection”	30
Price or Cost	Referred to cheap, price, cost, or bulk shopping	“cheaper than X store” “great prices” “stuff in bulk”	31
Quality and Freshness	Referred to the quality or freshness of products, including words like “best” and “good”	“quality of the products” “good produce” “fresher than X” “premium groceries”	33
Geographic Location	Referred to proximity or closeness to home, schools, work, or other shopping locations	“the only grocery store nearby” “close to my work” “proximity” “right by my house” “used to be close to my mother in law’s house” “I have no choice, I am forced to go to this one because there’s nothing else” “in my neighborhood”	43
Selection	Referred to the breadth of stuff offered at the store, selection, or variety. Includes comments about organic selection	“it has everything I want” “has a good variety of foods” “has things I can’t get elsewhere” “the selection is unmatched” “highest percentage of organic foods”	38
Social Reasons	Either referring to the social nature of a specific location (staff friendliness, social gathering spot) or to a social movement that is supported by choosing this store (*i.e.*, organic, local)	“I know the vendors and it is tradition to go here” “it is entertaining” “the people there are more welcoming” “I want to support a local Philadelphia institution” “I want to encourage the existence of this type of store in this type of neighborhood” “I like supporting local agriculture and knowing there is no intermediary between me and the farmer” “it’s a social place to see my neighbors”	25
Miscellaneous	Anything that did not fit into the other 6 categories. Usually item, individual or store specific	“it is open 24 hours a day” “I like their soft pretzels while I shop” “I only shophere if I’m making a recipe and forgot an ingredient” “someone else drives and chooses the store” “I like the layout, it is fast in and fast out” “my family members have dietary restrictions and this store has products that they can eat”	38

* Note: Answers can bridge more than one category.

### 3.3. Distance Traveled

**Figure 1:**
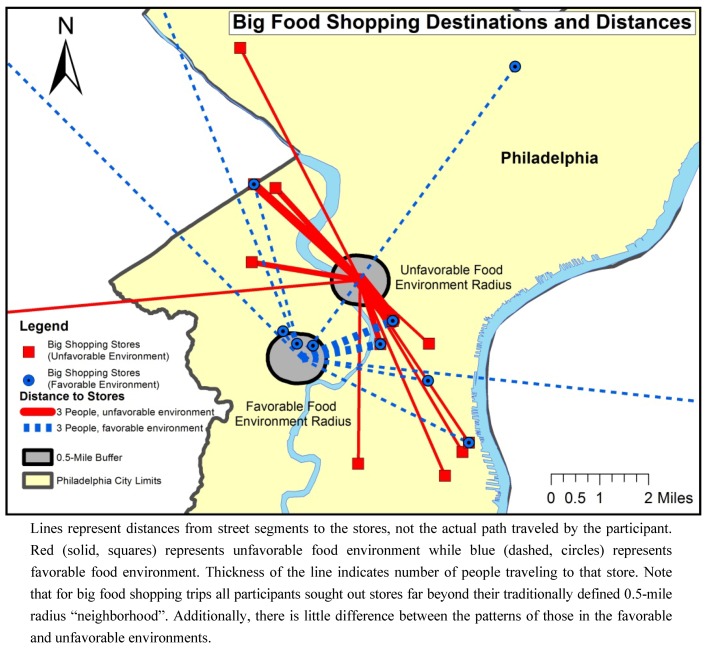
Euclidean distance between the favorable and unfavorable food environment street segments and food stores for big food shopping trips.

**Figure 2:**
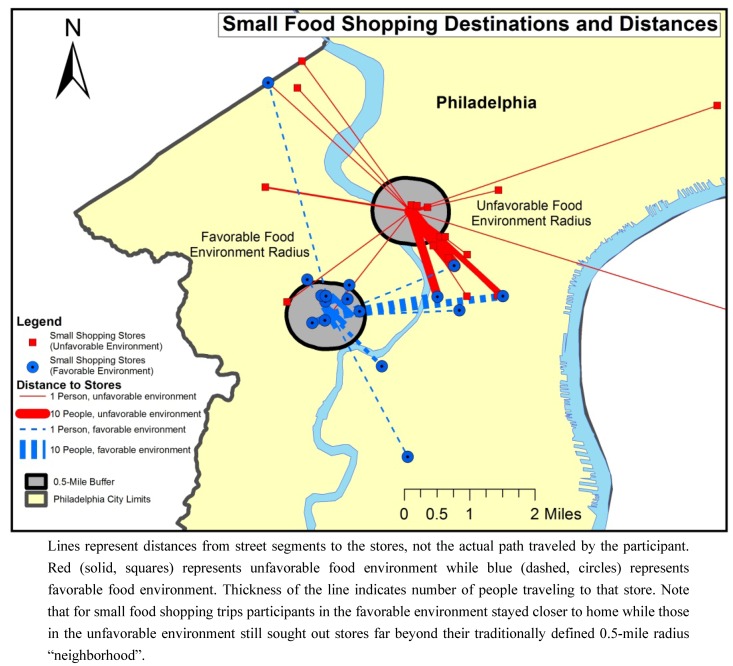
Euclidean distance between the favorable and unfavorable food environment street segments and food stores for small food shopping trips.

Mapping the location of food stores and the distance traveled by participants from each block, for big and small food shopping trips, revealed distinct patterns between the blocks based on the type of food shopping (see Figure 1 and Figure 2).

Participants traveled a mean of 3.0 miles (median 2.1 miles) for big food shopping trips, with no statistical difference in distance between the two blocks (*p *= 0.3). Participants in the favorable food environment traveled an average of 3.6 miles (median 1.8) for big trips while participants from the unfavorable environment traveled 2.5 miles (median 2.4 miles). Differences in the means may be attributable to outliers who chose to shop at specific stores not located within Philadelphia County due to brand preference or employment location (note blue lines in Figure 1). Of the 38 households undertaking big food shopping trips, 33 (87%) traveled outside of the traditional 0.5-mile radius used to designate local food environment. Overall, significantly more individuals in the favorable environment stay within the 0.5-mile radius for big food shopping trips than in unfavorable food environment (*p *= 0.02). Only 5 (28%) participants in the favorable and 5 (24%) in unfavorable environment did their big food shopping at the closest major supermarket to their home.

Participants traveled a mean 1.1 miles (median 0.9 miles) for small food shopping trips, and the difference between the two blocks was statistically significant (*p* < 0.01). Participants in the favorable food environment traveled a mean of 0.7 miles (median 0.4 miles) for small trips, while individuals from the unfavorable environment traveled a mean of 1.5 miles (median 1.4 miles) respectively. For small food shopping trips, 44% (n = 50) of the stores visited by participants were outside of the 0.5-mile radius. Overall, 88% (n = 45) of those stores visited by participants in the unfavorable environment were outside the 0.5-mile buffer, while 72% (n = 44) of those visited by the participants in the favorable environment remained within this boundary (*p* < 0.0001).

Of the stores identified by participants in the favorable environment for small food shopping trips, 28 (46%) were to the closest supermarket while only 13 (25%) of small trips by participants in the unfavorable environment were to the closest major supermarket.

### 3.4. Multivariate Factors in Distance Traveled

Several factors influenced the distance that participants traveled for food shopping including employment, car ownership, education, income and family size. Compared with full-time employment status, being a student increased the distance traveled for big food shopping trips by 7.9 miles (*p *= 0.0008), while being employed part-time increased the distance traveled by 5.3 miles (*p *= 0.006). Compared to having only one car, having more than one car increased travel distance by 2.5 miles (*p *= 0.05). 

Compared to those with a bachelor’s degrees, those with some college or an associate’s degree traveled 5.1 miles farther (*p *= 0.004). Compared to those making $100,000 or more per year, those making $50,000 to $75,000 traveled 0.7 miles farther (*p *= 0.07) while those making less than $20,000 per year traveled 5.6 miles less (*p *= 0.006). Every additional family member decreased distance traveled for big food shopping trips by 0.8 miles (*p *= 0.05).

For small food shopping trips, having more than one car increased distance traveled by 0.6 miles (*p *= 0.03). Compared to those with a bachelors, those with some college or an associate’s degree traveled 0.9 miles farther (*p *= 0.08). Making less than $20,000 was associated with a decrease in trip distance by 0.7 miles (*p *= 0.06) compared to those who make $100,000 or more per year. Being in a block with many food options nearby (favorable food environment) decreased distance traveled for small food shopping by 0.8 miles (*p *= 0.003) compared to those who have few to no food options nearby (unfavorable food environment).

## 4. Conclusions

This study sheds light on the intricate food shopping patterns of individuals within the context of disparate food environments. While participants recognized their traditionally defined food environments, they were not restricted to them. Differences were found between big and small food shopping trips, suggesting that the mechanisms by which the food environment influences eating habits may be context specific. A distinct dichotomy emerged for trips executed by car compared to those by foot. Simultaneously, individual factors including sociodemographics and personal preference influenced participants’ food shopping patterns.

Individuals from both blocks had perceptions of their food environment that matched the GIS-defined food environment. Yet regardless of the perceived environments, and consistent with previous literature on activity spaces [18,25], stores visited for both big and small food shopping trips were often beyond the 0.5-mile radius traditionally used to represent the extent of an individual’s food environment in an urban area. A majority of big trips were outside of the 0.5-mile radius and only a small fraction was to the store closest to participants’ homes. This is consistent with previous literature [32] that found a mean travel distance of 6.3 miles to superstores for food. Additionally, the finding that individuals travel beyond their residential food environment may help to explain mixed findings from quasi-experimental studies evaluating eating habits after the addition of a new retail food outlet [33,34,35]. Qualitative answers about store choice suggest that big food shopping trips are more likely to be made as separate errands, using a car for transportation, and to seek out bulk items at lower prices. With small trips, however, those in the food environment with many options traveled shorter distances and were more likely to use their closest store. The greater importance of geographic proximity for small food shopping trips relative to big food shopping trips represents a distinction not currently recognized in the literature. It is possible that once an individual has made the decision to drive, possibly due to size of trip, distance becomes less important and other factors, such as quality and prices, become more important. Therefore, the scale of the food environment that is appropriate may be altered based on both transportation mode and shopping trip size. As has been noted elsewhere [33], future research should consider the complexities of shopping trip size, store choice, and transportation mode when measuring the food environment.

Material and social resources may play a key role in understanding shopping behavior and the effects of the built food environment. The similarity between blocks in frequency of trips and priorities for choosing stores indicate that factors other than geographic proximity are influential in food store choice. In addition, differences in the distance traveled were explained in part by individual and household characteristics including car ownership, employment status, education, and income levels. Due to the fact that this study was performed in middle- to upper-income locations, these findings may highlight the ability of individuals to seek out healthy options if they have the financial means. For example, those who perceive that they have fewer options around them may utilize resources (such as car ownership or income) to counteract this local deficiency. Since study participants were predominantly white and middle class, the availability of resources to compensate the geographic contrast may help explain some of the similarities in shopping patterns between blocks. However, in populations with fewer resources in regard to car ownership, employment status, educational attainment and household income, the local food environment may play a more salient role in their food shopping patterns. Previous research supports this notion, suggesting that the size of an individual’s activity space is influenced by sociodemographic characteristics [18,25]. Further research should aim to investigate the relationships discussed here across different socioeconomic groups.

Qualitative research regarding food store choice helped to shed light on some of the possible reasons individuals travel outside of their neighborhood and in such different patterns from their neighbors with whom they share a food environment. Emphasis on convenience and geographic location show that the built environment is still of importance but that the mechanisms between the built food environment context and health are more complex than assumed. While many individuals referenced convenience, the meaning of this to residents is not entirely clear. Future research is needed to better understand the concepts of convenience and proximity in the context of food shopping. Consideration for the multiple contexts in which people live, work, and play has been identified as an important research aim [30,33,36], however, with the exception of a few studies [37,38,39,40], current research is still limited. Qualitative GIS research methods that are designed to elicit the meaning of geographic relationships, such as geo-ethnography [41], may help researchers understand the interplay between various motivations behind store choice. Discussions about geographic proximity to a variety of locations including children’s schools, elder relative’s homes, or work indicate that future studies cannot assume a participant’s home as the starting location of a food environment. Few studies investigate the relationship between non-residential food environments and health [37,42]. Factors in food store choice other than proximity, such as food price [43,44,45], quality [46,47], and selection [48], need to be measured within the context of individual preference. Mixed methods approaches may shed more light on the role of store choice in shopping patterns and ultimately health behaviors.

This study has a number of limitations. The small sample size and lack of diversity within the sample limits our ability to investigate broader trends, perform more complex statistical analyses, or generalize the findings beyond middle and upper-income white households in Philadelphia. The lack of health behavior data restricts the ability of this study to address how shopping patterns may subsequently affect health. Additionally, the mismatch of age and student status between the blocks prevents a perfect comparison and analysis of shopping behavior as younger students may use different transportation modes or visit different food stores. A lower contact and response rate may result in nonresponse bias, although participants had similar sociodemographic characteristics as those recorded in Census 2000 for the block groups that contain these street face blocks and were similar to other door-to-door research on the same topic [49]. Finally, due to the lack of previous research in this area no data exists on the validity and reliability of the survey instrument used. However, this study raises a number of questions about the current methods used to measure the food environment or how we conceptualize the link between the food environment and health behaviors. It identifies a number of complications to traditional buffer definitions of the food environment while the use of qualitative, open-ended questions gives a more comprehensive understanding of the decisions individuals make within their environments. The mixed methods used in this research may be more feasible for a local government or public health agency to implement due to their lower cost and level of participant commitment respective to GPS or travel diary methods of activity space. As the field moves forward, discussions are necessary to determine how we view the role of individual choice or preference. While much progress has been made towards building a body of evidence that supports the neighborhood effects, less attention has been paid to the assumptions underlying our current measurement of the built environment or to carefully interpreting findings within a theoretical framework. Advancement of food environment research depends upon gaining an understanding of how individuals interact with, and move within, the built environment.
